# Single‐cell RNA sequencing reveals the heterogeneity of MYH11+ tumour‐associated fibroblasts between left‐sided and right‐sided colorectal cancer

**DOI:** 10.1111/jcmm.70102

**Published:** 2024-09-18

**Authors:** Chao Wang, Yue Zhao, Sainan Zhang, Meiyu Du, Guanzhi He, Senwei Tan, Hailong Li, Duoyi Zhang, Liang Cheng

**Affiliations:** ^1^ College of Bioinformatics Science and Technology Harbin Medical University Harbin Heilongjiang China; ^2^ The 2nd Affiliated Hospital of Harbin Medical University, Harbin Medical University Harbin Heilongjiang China; ^3^ NHC Key Laboratory of Molecular Probe and Targeted Diagnosis and Therapy Harbin Medical University Harbin Heilongjiang China

**Keywords:** colorectal cancer, left‐sided and right‐sided colorectal cancer, single cell

## Abstract

Colorectal cancer (CRC) exhibits considerable heterogeneity on tumour location. However, there is still a lack of comprehensive annotation regarding the characteristics and differences between the left‐sided (L‐CRC) and right‐sided (R‐CRC) CRC. Here, we performed single‐cell RNA sequencing (scRNA‐seq) on immune and stromal cells from 12 L‐CRC and 10 R‐CRC patients. We found that L‐CRC exhibited stronger tumour invasion and poor prognosis compared with R‐CRC. In addition, functional enrichment analysis of a normal cohort showed that fibroblasts of left colon are associated with tumour‐related pathways. This suggested that the heterogeneity observed in both L‐CRC and R‐CRC may be influenced by the specific location within the colon itself. Further, we identified a potentially novel MYH11+ cancer‐associated fibroblast (CAF) subset predominantly enriched in L‐CRC. Moreover, we found that MYH11+ CAFs may promote tumour migration via interacting with macrophages, and was associated with poor prognosis in CRC. In summary, our study revealed the crucial role of MYH11+ CAFs in predicting a poor prognosis, thereby contributing valuable insights to the exploration of heterogeneity in L‐CRC and R‐CRC.

## 1 INTRODUCTION

Colorectal cancer (CRC) ranks as the third most prevalent cancer globally, resulting in approximately 900,000 deaths every year.^1^, ^2^ Classified by anatomical locations, CRC can be classified into two subtypes: left‐sided (L‐CRC) and right‐sided (R‐CRC) CRC. Clinical data highlight that approximately 63% of CRC patients are diagnosed with L‐CRC,^3^ indicating a higher incidence of tumours in the left colon. Moreover, notable differences in prognosis and treatment outcomes have been noted between L‐CRC and R‐CRC.^4^, ^5^, ^6^ However, the lack of comprehensive molecular studies has hindered our understanding of the underlying mechanisms contributing to the divergence between L‐CRC and R‐CRC.

Single‐cell RNA sequencing (scRNA‐seq) has emerged as a powerful technology to uncover cellular characteristics in diseases, particularly cancers.^7^, ^8^, ^9^ In the context of CRC, scRNA‐seq analyses have revealed the heterogeneity of the tumour microenvironment (TME), offering insights into diverse cell types like cancer‐associated fibroblasts (CAFs) and myeloid cells.^8^, ^10^, ^11^ Both CAFs and myeloid cells are abundantly enriched in the TME and essential for formation and metastasis of cancer cells.^12^, ^13^ But to date, there is a lack of comprehensive understanding regarding the heterogeneity between L‐CRC and R‐CRC at the molecular level.

To elucidate the molecular heterogeneity at the cellular level, we investigated the roles of specific cell types in L‐CRC and R‐CRC based on scRNA‐seq analysis. Firstly, 12 L‐CRC samples, 10 R‐CRC samples and a normal control cohort were collected to construct a single‐cell atlas. Subsequently, comprehensive transcriptomic characterizations were descripted for L‐CRC and R‐CRC. Then, functional enrichment and cell–cell interaction analyses were performed to target key cells with different levels of communication. Finally, a previously unidentified MYH11+ CAF subset was characterized and verified to be associated with a poorer prognosis. Our results provides new insights into the relationship between the location and severity of CRC, and offers new directions for precise treatment and prognosis prediction.

## 2 MATERIALS AND METHODS

### 2.1 Single‐cell RNA data source and preprocess

Single‐cell data of CRC patients were collected from GEO database (GSE132465, GSE188711).^9^, ^14^ Normal colon samples, including both left normal samples (L‐N) and right normal samples (R‐N), were obtained from Single Cell Portal (SCP259).^15^ The generated outputs were processed using the Seurat package (version 4.0.5).^16^ To filter out low‐quality cells, the following criteria were adopted. (1) Cells with few (<200) or too much genes (>6000); (2) cells with over 25% of mitochondrial genes.

### 2.2 Dimension reduction and clustering analysis

The ‘SCTransform’ method was used to standardize the data to reduce the difference in the sequencing depth of cells in each sample. To eliminate the batch effect, the ‘Harmony’ function was used to eliminate the batch correction before cluster analysis. Then, the ‘FindNeighbors’ and ‘FindCluster’ functions were applied to obtain the cell clusters, and the ‘FindAllMarkers’ function was used to identify the marker genes of each cluster. According to the marker genes, all cells of the datasets were divided into non‐immune cells and immune cells. Non‐immune cells included epithelial cells (EPCAM, CDH1), fibroblast cells (DCN, COL3A1) and endothelial cells (ENG, PECAM1). Immune cells included myeloid cells (FCGR3A, CD86), T cells (CD3D, CD3E) and B cells (CD79A, MS4A1).

### 2.3 Differential expression analysis

The ‘FindMarkers’ function in was used to identify differentially expressed genes (DEGs) on the left and right sides of the colon in CRC and normal control groups. Significantly different genes (Padj <0.05 and | log_2_FC | >1) were selected for downstream analysis.

### 2.4 Functional enrichment analysis

Significantly differentially expressed genes of different groups were used for gene set enrichment analysis by clusterProfiler package (version 4.0.1),^17^ including Gene Ontology (GO) function and KEGG pathway enrichment analysis. The main signal transduction pathways were identified through quantitative analysis.

### 2.5 Calculate of DNA damage, proliferation, stemness and hypoxia scores

The immune cell infiltration in tumour tissue could be predicted by utilizing pre‐screened matrix related gene sets and immune related gene sets in samples. The stroma scores could be evaluate by the level of stroma cell infiltration in tumour tissue. The immune scores could be evaluate by the level of immune cell infiltration in tumour tissues. The DNA damage, tumour proliferation, stemness and hypoxia scores could be calculated by using genes related to cancer functional statuses. The gene sets were collected from the CancerSEA database.^18^

### 2.6 Cell–cell interaction analysis

The CellPhoneDB software (version 3)^19^ was used to evaluate ligand–receptor pairs between all cell types of L‐CRC and R‐CRC, and significantly enriched (*p* value <0.05) ligand–receptor pairs were selected for downstream analysis. In addition, the cell interaction network diagram of CRC was constructed using the R package ‘circle’ to illustrate the regulatory relationship between cells, and a bubble plot was used to display the interaction pairs.

### 2.7 Cell developmental trajectory

Pseudotime analysis was conducted to predict the evolutionary trajectory by the Monocle package (version 2)^20^ based on the key genes of cell clusters. The CellDataSet object was created with the parameter ‘expressionFamily = negbinomial’. Then the cell differentiation trajectory was inferred with the default parameters of Monocle after dimension reduction and cell ordering.

### 2.8 Survival analysis

The Kaplan Meier survival curve of OS based on the expression of the top 10 characteristic genes in MYH11+ cell clusters was performed using the online bioinformatics tool Kaplan–Meiser plotter.^21^

## 3 RESULTS

### 3.1 High‐resolution scRNA‐seq reveals the global landscape of L‐CRC and R‐CRC

**FIGURE 1:**
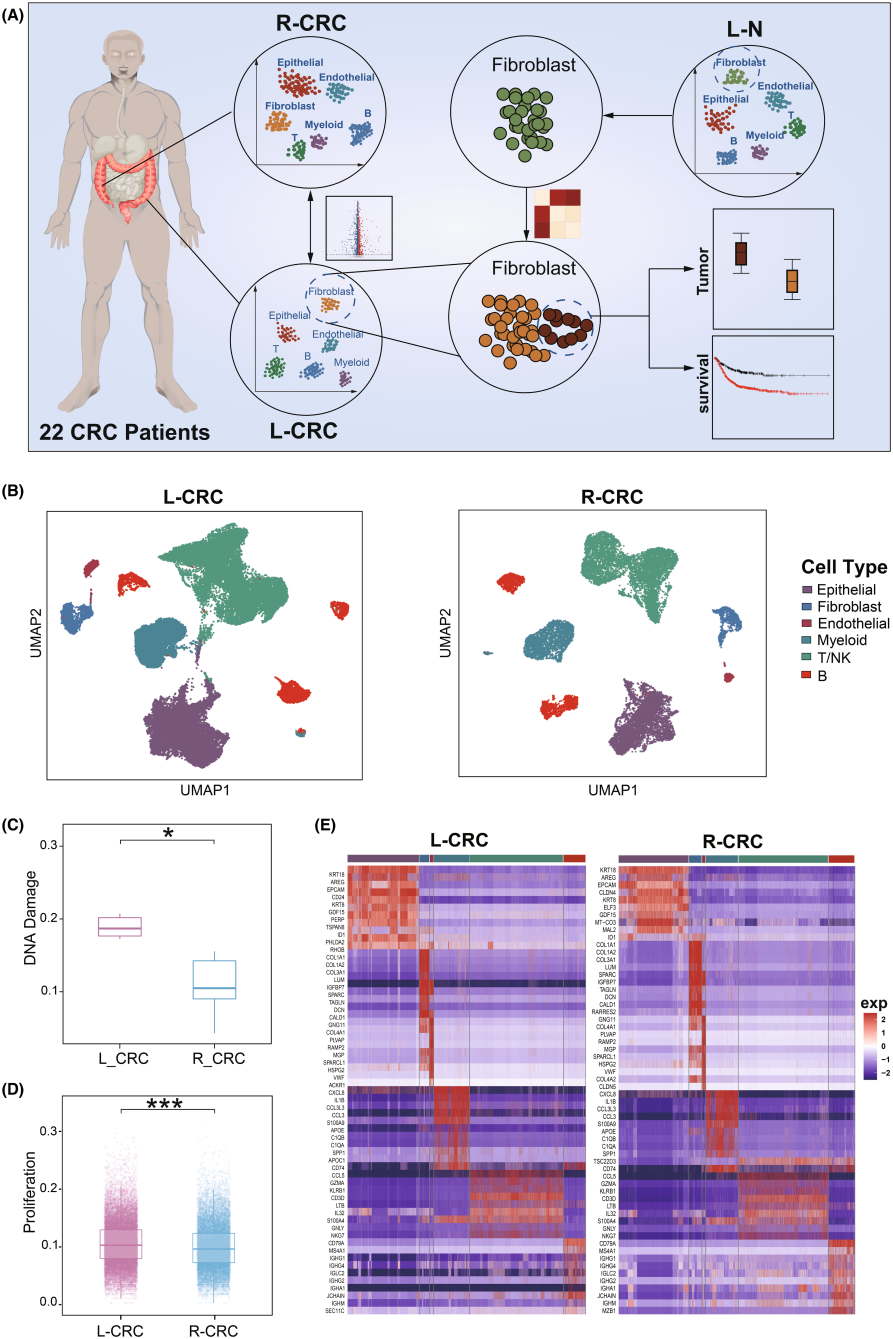
Construction of single‐cell atlas in L‐CRC and R‐CRC patients. (A) Workflow diagram of the study. (B) UMAP plot showing the cell types of L‐CRC and R‐CRC. (C) Barplot showing DNA Damage score of L‐CRC and R‐CRC, *p < 0.05, by *t*‐test. (D) Barplot showing the proliferation score of L‐CRC and R‐CRC, with dots representing cells, ***p < 0.001, by *t*‐test. E. Heatmap showing highly expressed genes across six cell types of L‐CRC and R‐CRC.

To characterize the immune landscape in L‐CRC and R‐CRC, we performed scRNA‐seq analysis on 22 CRC samples (Figure 1A, Table S1), including 12 samples from L‐CRC and 10 samples from R‐CRC. Through data quality control of the samples, a total of 41,155 high‐quality cells were obtained and sorted into 6 cell types (T/NK cells, B cells, myeloid cells, epithelial cells, fibroblasts and endothelial cells) according to previously marker genes (Figure 1B and Figure S1A, S1B).

To avoid the influence of significant cell numbers differences on the results, we conducted a *t*‐test to assess the cell count bias on both sides samples and observed no significant difference (*p* = 0.65) (Figure S1C). Subsequently, we calculated DNA damage scores to assess potential mutation accumulation in L‐CRC and R‐CRC samples, it was observed that the L‐CRC showed higher scores than R‐CRC, indicating a higher degree of severity in L‐CRC patients (Figure 1C). We further calculated the proliferation scores by collecting genes related to cancer proliferation functional status from the canserSEA database,^18^ the results showed that the cells on L‐CRC had greater proliferation ability, which may be related to the tumour invasion and development (Figure 1D). Next, to explore biological significance of transcriptional changes between L‐CRC and R‐CRC, we analysed the top 10 highly expressed genes across different cell types (Figure 1E). We found that compared with immune cells, non‐immune cells had more pronounced transcriptional differences on both sides of CRC. For example, RHOB, which has been shown to control cell growth, differentiation, adhesion and migration in the tumour microenvironment,^22^ is highly expressed in the fibroblasts of L‐CRC. The other gene PHLDA2, which has been reported to be associated with lymph node metastasis and TNM staging,^23^ had a higher expression level in epithelial cells of L‐CRC compared to R‐CRC. Taken together, our scRNA‐seq analysis dissected the differences of the landscape between L‐CRC and R‐CRC, and showed that L‐CRC might obtain higher tumour heterogeneity than R‐CRC. And the heterogeneity is more obvious in non‐immune cells.

### 3.2 Fibroblasts of both L‐N and L‐CRC promote tumour progression

**FIGURE 2:**
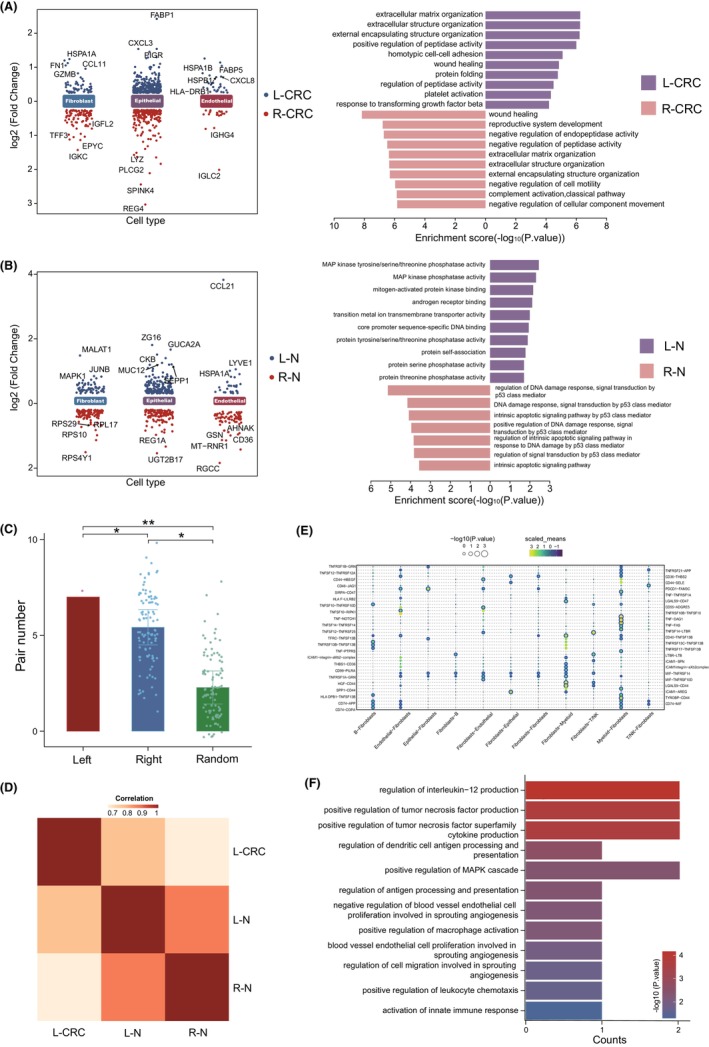
Characterization of fibroblasts in both sides of colon for patients and healthy controls. (A) Symmetric scatter plot showing the upregulated genes in non‐immune cells of L‐CRC and R‐CRC (left), and barplot showing differential pathways enriched in fibroblasts of L‐CRC and R‐CRC by GO (right). (B) Symmetric scatter plot showing the upregulated genes in non‐immune cells of L‐ and R‐N (left), and barplot showing differential pathways enriched in fibroblasts of L‐ and R‐N by GO (right). (C) Scatter barplot showing the number of protein interactions pairs across three groups. *p < 0.05, **p < 0.01, by *t*‐test. (D) Heatmap showing the correlations among L‐CRC, L‐ and R‐N fibroblast. (E) Bubble heatmap showing the mean strength for ligand–receptor interaction pairs between fibroblast cells (L‐CRC) and other cell types. Dot size indicates *p*‐value, and coloured by the mean value of the average ligand–receptor pair expression in cell types. (F) Functional enrichment showing specific cellular communication processes in fibroblast of L‐CRC.

Given that location characteristics determine the cellular composition of tissues,^24^ we further evaluated the relationships between tissue (left and right colon) characteristics and non‐immune cell differences. To provide a more detailed insight of cellular features in both sides of CRC, the differentially expressed genes of all cell types were detected. The results showed that there was a large difference in the percentage of significant upregulated genes of non‐immune cells between L‐CRC and R‐CRC (Figure S1D). In addition, functional enrichment analysis revealed that tumour progression‐related pathways were significantly activated in fibroblasts of L‐CRC compared with those of R‐CRC (Figure 2A). For example, fibroblasts in L‐CRC showed a high activation of response to transforming growth factor beta (TGF‐β), which could lead to extracellular stroma deposition and CAFs formation, resulting in fibrotic disease and cancers.^25^ For the other two non‐immune cell types, there were few tumour‐related functional differences between L‐CRC and R‐CRC (Figure S1E). Specifically, epithelium in L‐CRC were mainly involved in RNA metabolic, while those in R‐CRC play the role of modification of proteins or organelles. In addition, endothelial cells are involved in regulation and activation of cell activities between L‐CRC and R‐CRC, respectively. Furthermore, we also found that the significant down‐regulated genes in non‐immune cells of L‐CRC and R‐CRC were enriched in some metabolic pathways, rather than tumour‐related pathways. Since tumours with different stages possessed distinct biological characteristics, we screened out samples with stage III tumour for enrichment analysis (Figure S2A). The results revealed that fibroblasts of L‐CRC specifically activated tumour‐related pathways compared with those of R‐CRC (Figure S2B), suggesting that fibroblasts of L‐CRC were closely related to tumour progression.

Fibroblasts in normal tissues may transform into CAFs under stimulating by cytokines such as TGF‐β, IL‐1 and IL‐6.^25^, ^26^ We further conducted scRNA analysis by importing another dataset to investigate whether fibroblasts exhibit significant differences on L‐ and R‐N. Functional enrichment analysis revealed that upregulated genes of L‐N activated MAPK signalling pathway (Figure 2B), which was closely related to cell proliferation, angiogenesis and lymphovascular invasion, and was a critical cause of various cancers.^27^, ^28^ In addition, tumour inhibit‐related signalling pathways were activated in fibroblasts from R‐N (Figure 2B). For example, the regulation of DNA damage response and signal transduction mediated by P53, a tumour suppressor protein and transcription factor that regulates cell division, prevents DNA mutated or damaged cells from dividing and conducts apoptotic signals to prevent tumour formation.^29^ In addition, we found that upregulated genes in endothelial and epithelial cells of L‐RN and R‐N did not exhibit antagonistic tumour‐related pathways similar to those in fibroblasts (Figure S2C). These results suggested that there were tumour‐related features in the fibroblasts of the left colon from both CRC patients and normal controls.

To emphasize the effect of colon location on the differences between L‐CRC and R‐CRC, we further evaluated the associations between fibroblasts of left colon from both CRC patients and normal controls. The protein interaction analysis revealed that fibroblasts of L‐CRC had more interacted pairs with those of L‐N (Figure 2C). In addition, the correlation analysis showed that the signature scores of fibroblasts from L‐CRC had a higher correlation with those from L‐N compared with R‐N (Figure 2D). Given that the molecular interactions mediates cell–cell interactions and biological function, we further calculated the attraction strengths of ligand–receptor pairs and identified hundreds of intercellular interaction pairs in both L‐CRC and R‐CRC (Figure S2D). The results showed that more tumour invasion‐related interactions were detected in fibroblasts of L‐CRC (Figure 2E). For example, of the ligand–receptor pairs pertaining fibroblasts to and myeloid cells, THBS1‐CD36, which has been reported to be associated with tumour growth and metastasis in CRC,^30^, ^31^ was significantly enriched in fibroblasts, indicating a potential role of fibroblasts from L‐CRC in promoting tumour progression. Moreover, the interactional genes from fibroblasts of L‐CRC were also involved in cancer progression (Figure 2F), such as positive regulation of MAPK signalling pathway. Collectively, our results suggested that fibroblasts from L‐CRC showed higher tumour‐promoting activity compared with L‐CRC.

### 3.3 CAF subtype of fibroblasts has tumour heterogeneity on L‐CRC and R‐CRC

**FIGURE 3:**
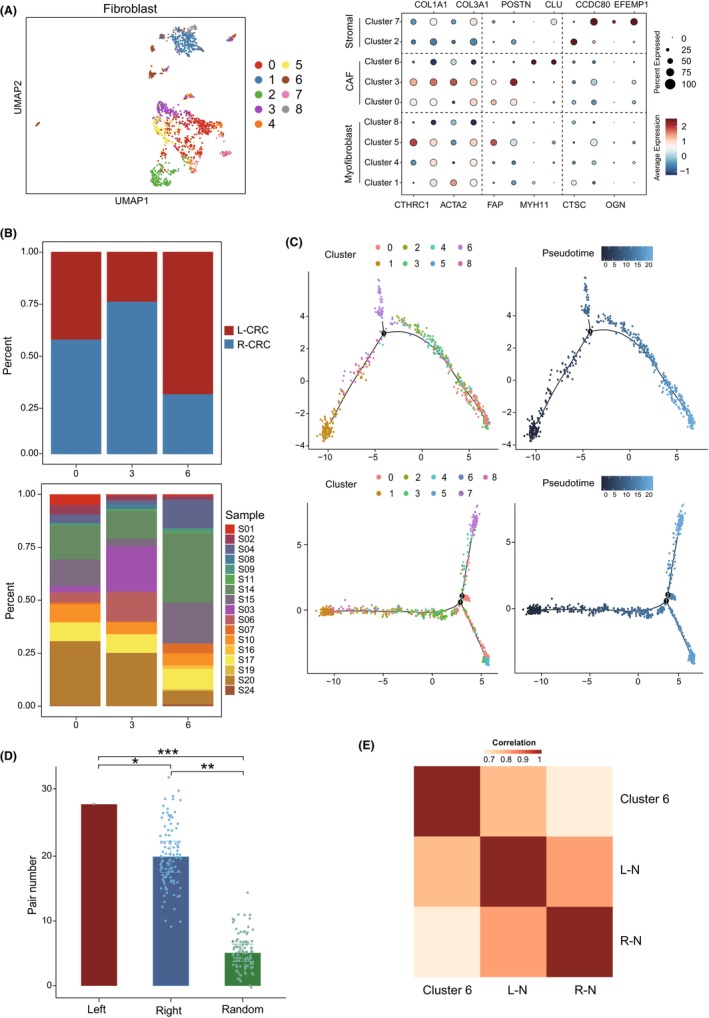
Identification of L‐CRC‐enriched CAF subset of fibroblasts. (A) UMAP plot showing the re‐clustered cell subsets of fibroblasts (left) based on the known marker genes (right). (B) Barplot showing the contribution of tissue location (top) and samples (bottom) in three CAF subsets. (C) Pseudotime trajectory showing the differentiation among cell subsets of L‐CRC (top) and R‐CRC (bottom), cells are coloured by cluster (left) or pseudotime (right). (D) Scatter barplot showing the number of protein interactions pairs across three groups. *p < 0.05, **p < 0.01, ***p < 0.001, by *t*‐test. (E) Heatmap showing the correlations of CAF cluster 6 and cell clusters of L‐ and R‐N.

CAFs exhibit high heterogeneity to exert opposite effects in tumour progression.^32^ To identify CAF subtypes that contributed to TME, we re‐clustered the fibroblasts in both side CRC according to their transcriptome profiles. In total, nine unique fibroblast clusters were identified (Figure 3A), among which clusters 0, 3 and 6 were identified as CAFs while clusters 1, 4, 5 and 8 were identified as myofibroblast, and the clusters 2 and 7 were identified as stromal cells (Figure 3A). Further analysis of these three CAF clusters showed that the cluster 6 was highly enriched in L‐CRC compared with others (Figure 3B). To detect whether there was sample bias in clusters, we further compared the distribution of samples in these three clusters, and the results showed that the samples were evenly distributed in each group (Figure 3B), suggesting that L‐CRC may had a higher density of infiltrated CAF cluster 6.

Given that the cell differentiation trajectory is of great significance to reveal the formation mechanism and treatment strategy of tumour,^33^ we further investigated the origin of cluster 6 in L‐CRC and R‐CRC. Pseudotime trajectory analysis of all fibroblasts in the dataset showed that CAF cluster 6 was transformed and activated from cluster 1 in both sides of CRC (Figure 3C). Notably, cluster 6 was the endpoint branch only in L‐CRC compared with R‐CRC. To investigate the underlying mechanism of this location‐specific changes, we conducted pathway enrichment analysis using key genes during differentiation. The results showed that the genes (RAMP1, MYLK and MYH11) of cluster 6 end‐stage were enriched with tumour activation pathways such as cell proliferation and angiogenesis (Figure S3A). For example, RAMP1, which encodes a protein that regulates cell proliferation and angiogenesis, had been reported to induce the functional depletion of CD8+ T cells with tumour invasion.^34^ The MYLK and MYH11 genes encode actin and myosin, which play an important role in the contraction and migration of tumour cells. John et al. found that CAFs with high expression of MYH11 exist in the early stage of lung cancer, suggesting that MYH11 may play an important role in the early development of tumours. Collectively, these genes that were highly expressed in CAF cluster 6 of L‐CRC may play important roles in the development and metastasis of tumours.

Since CAF cluster 6 was enriched in L‐CRC and associated with the increased with elevated tumour‐related genes, we further evaluated the relationship between CAF cluster 6 and normal colon on both sides to explore why L‐CRC fibroblasts showed stronger tumour associations. The protein interaction analysis in scRNA‐seq showed that CAF cluster 6 mostly interacted with cells in L‐N compared with R‐N (Figure 3D). In addition, we found a significantly positive correlation between the signature score of CAF cluster 6 and cells of L‐N (Figure 3E), indicating that CAF cluster 6 might play a vital role by interacting with cells in L‐N. In summary, these data suggested that CAF cluster 6 was closely associated with tumour development and WAS more likely to interact with cells in the left colon (including both normal tissue and tumours).

### 3.4 MYH11+ CAFs are associated with poor prognosis

**FIGURE 4:**
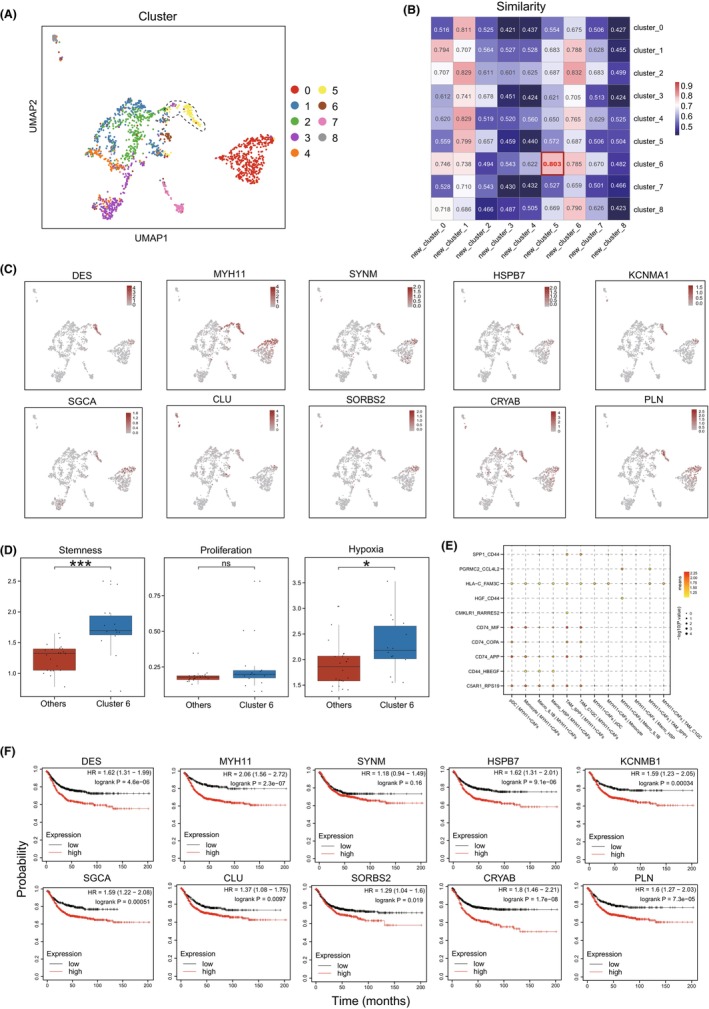
Functional description of MYH11 + CAFs. (A) UMAP plot showing the re‐clustered subsets of fibroblasts in validation dataset (GSE188711). (B) Similarity of fibroblast subsets from two datasets. (C) UMAP plot showing the expression of the top 10 highly expressed genes (CAF cluster 6) in fibroblasts of validation dataset (GSE188711). (D) Barplot showing tumour stemness tumour proliferation score and tumour hypoxia scores of CAF cluster 6 and other clusters, with dots representing samples, ^ns^ *p* > 0.05, *p < 0.05, ***p < 0.001, by *t*‐test. (E) Bubble heatmap showing the mean strength for ligand–receptor interaction pairs between MYH11+ CAFs and subtypes of myeloid cells. Dot size indicates *p*‐value, and coloured by the mean value of the average ligand–receptor pair expression in cell types. (F) Survival analysis of the top 10 highly expressed genes in MYH11+ CAFs.

To confirm that the presence of CAF cluster 6 in L‐CRC is not an incidental occurrence, the other independent scRNA‐seq dataset was collected and analysed. We found that the cluster 5 of re‐clustered fibroblasts in the validation dataset was enriched in L‐CRC (Figures 4A and S3B). In addition, the signature score of the cluster 5 had a significantly positive correlation with that of CAF cluster 6 (Figure 4B). Moreover, the cluster 5 also highly expressed top genes of CAF cluster 6 (Figure 4C), indicating that the cluster 5 could well define a CAF subset, which was highly similar to CAF cluster 6. These results also suggested that CAF cluster 6 may be widespread in L‐CRC.

Given that CAF cluster 6 may play an important role in L‐CRC, we then searched for markers to identify CAF cluster 6. The characteristic gene analysis revealed top 10 upregulated markers distinguished CAF cluster 6 from other fibroblast subsets (Table S2). Among them, MYH11 had a significant enrichment in CAF cluster 6 than other subsets compared with other genes. In addition, we found that MYH11 was mainly expressed in CAFs and could well define CAF cluster 6 in our scRNA‐seq dataset (Figure S3C). Moreover, MYH11 had a higher expression level on L‐CRC than R‐CRC in CAF cluster 6 (Figure S3D). These results demonstrate that MYH11+ CAFs represent as a specific CAF subset in L‐CRC.

To evaluate the function of MYH11+ CAFs in tumour progression of L‐CRC, we calculated tumour‐related scores in scRNA‐seq of L‐CRC to reveal that MYH11+ CAFs had elevated expression of higher proliferation, stemness and hypoxia scores than other subsets (Figure 4D). Moreover, the cell interaction analysis showed that MYH11+ CAFs mostly interacted with subtypes of myeloid cells (Figure 4E). Among the interaction molecular pairs, tumour invasion‐related pairs, including CD74_MIF, SPP1_CD44, were enriched among macrophages and MYH11+ CAFs. Importantly, functional enrichment analysis revealed that muscle‐related pathways were significantly activated in MYH11+ CAFs, suggesting that MYH11+ CAFs may contributes to the migration and attachment of tumour cells to normal tissue (Figure S3E). For example, the protein encoded by MYH11 was involved in muscle contraction and the function of vascular smooth muscle cells, Pia et al. found that MYH11 mutations contribute to human intestinal tumours, and that upregulated MYH11 may affect cell energy balance or interfere with cell lineage decisions in tumour progenitors.^35^, ^36^ Moreover, survival analysis showed that higher levels of markers infiltration in MYH11+ CAFs were significantly associated with worse outcome in CRC (Figure 4F). Collectively, these results indicated that MYH11+ CAFs were associated with the poor prognosis of patients with CRC, and may be considered as potential therapeutic target cells for CRC.

## 4 DISCUSSION

The discrepancy in the colon's embryonic development results in variations in its eventual position in the body.^37^ Tumours located on the right or left side of the colon display unique biological features, leading to differing clinical presentations and treatment approaches, indicating the existence of specific oncogenic factors in different locations. For example, researchers found that TP53 mutation, which leads to the loss of normal DNA damage repair and apoptosis regulation functions, are more prevalent in L‐CRC and associated with poorer prognosis,^38^ whereas BRAF mutation occur more frequently in R‐CRC.^39^, ^40^ In addition, biomarkers like CpG island methylation which are more prevalent in R‐CRC, are associated with the effectiveness of immunotherapy and linked to a more favourable prognosis.^40^, ^41^, ^42^ Nevertheless, the differences between L‐CRC and R‐CRC had not been fully elucidated at the molecular level.^39^ Here, we constructed a compendium of a high‐resolution single‐cell landscape and identified cellular characteristics for both L‐CRC and R‐CRC. Further, we calculated the DNA damage and proliferation scores of L‐CRC and R‐CRC samples, and found that L‐CRC exhibited a higher degree of severity compared to R‐CRC. Additionally, we also found that tumour‐related pathways, such as cell proliferation, invasion and metastasis were specific enriched in fibroblasts of L‐CRC, suggested a distinctive pattern of tumour progression of fibroblasts in L‐CRC. Conversely, the activation of tumour suppressor pathways may contribute to the relatively better prognosis and lower aggressiveness observed in these cases with R‐CRC. These findings emphasized the importance of considering the molecular heterogeneity between L‐CRC and R‐CRC for prognostic evaluation.

L‐CRC has been associated with a more aggressive phenotype, higher tumour stage, and poorer clinical outcomes compared to R‐CRC.^43^ For example, for four consensus molecular subtypes (CMS) of CRC, L‐CRC is more enriched in CMS3 and CMS4 than R‐CRC. Among them, CMS4 has the worst overall survival among all stages.^39^ The molecular features identified in this study may contribute to explaining the observed clinical differences. Furthermore, the tumour‐related signal pathways of fibroblasts observed in L‐CRC indicated a potential influence on tumour progression and highlights the importance of the tumour microenvironment in modulating tumour behaviour. Fibroblasts play a crucial role in tumour‐stromal interactions, extracellular matrix remodelling and angiogenesis.^44^ In our study, fibroblasts exhibit not only significant differences between L‐CRC and R‐CRC but also functional disparities in both L‐ and R‐N. We found that fibroblasts in L‐N were enriched in tumour‐associated pathways. This suggested that the fibroblast population in L‐N may already possess a predisposition towards tumour‐related functions. Moreover, we identified a MYH11+ CAF subtype within fibroblasts that was enriched in left‐sided CRC, associated with high tumour scores, and poor prognosis. This specific CAF could potentially serve as a target cell type for future targeted therapies.

Our study still has certain limitations. First, the dataset of normal colon might have a restricted sample size and restricted the comprehensive characterization of the microenvironment. It is important to note that the normal samples used in this study do not cause a bias impact on the biological significance. Our choice of normal samples was intended to explore the relationship between the functional status of the left and right colorectal regions under normal circumstances and disease severity. Second, the potential relationships between the fibroblasts and myeloid cells in L‐N were not fully investigated to explain the underlying mechanism. Third, the mouse modes were not engineered to verify specific functions of MYH11+ CAFs. Therefore, further in‐depth in vivo and in vitro experimental explorations are necessary to determine the actual clinical value of our results.

## 5 CONCLUSIONS

In summary, our scRNA‐seq analysis revealed distinct molecular characteristics of L‐CRC and R‐CRC. The severity difference, fibroblast enrichment in tumour‐related pathways, and differential activation of tumour suppressor pathways provide molecular insights into the disparities observed between these two tumour types. MYH11+ CAFs induce tumour invasion through interacting with myeloid cells. Our findings provide comprehensive insight into the TME heterogeneity of L‐CRC and R‐CRC, and emphasize the importance of MYH11+ CAFs, which may be considered as a potential therapeutic target cells for CRC.

## AUTHOR CONTRIBUTIONS

**Chao Wang:** Data curation (equal); formal analysis (equal). **Yue Zhao:** Data curation (equal); formal analysis (equal); writing – original draft (lead). **Sainan Zhang:** Writing – review and editing (lead). **Meiyu Du:** Writing – review and editing (supporting). **Guanzhi He:** Validation (equal). **Senwei Tan:** Visualization (equal). **Hailong Li:** Visualization (equal). **Duoyi Zhang:** Visualization (equal). **Liang Cheng:** Conceptualization (lead).

## FUNDING INFORMATION

This work was supported by the Tou‐Yan Innovation Team Program of the Heilongjiang Province (2019‐15) and the National Natural Science Foundation of China (62222104, 62172130).

## CONFLICT OF INTEREST STATEMENT

The authors confirm that there are no conflicts of interest.

## Supporting information


Figure S1.

Figure S2.

Figure S3.

Table S1.

Table S2.

## Data Availability

The data used in this study are from public available datasets. Single‐cell data of CRC patients were collected from GEO database (GSE132465, GSE188711). Normal colon samples, including both left normal samples and right normal samples, were obtained from Single Cell Portal (SCP259). The information is detailed in the Section 2, and do not involve ethical approval.
