# Supplementary figures and images for: Single‐cell RNA sequencing reveals the heterogeneity of MYH11+ tumour‐associated fibroblasts between left‐sided and right‐sided colorectal cancer
Source: J Cell Mol Med. 2024 Sep 18;28(18):e70102. doi: 10.1111/jcmm.70102 (PMC11410558; doi:10.1111/jcmm.70102)

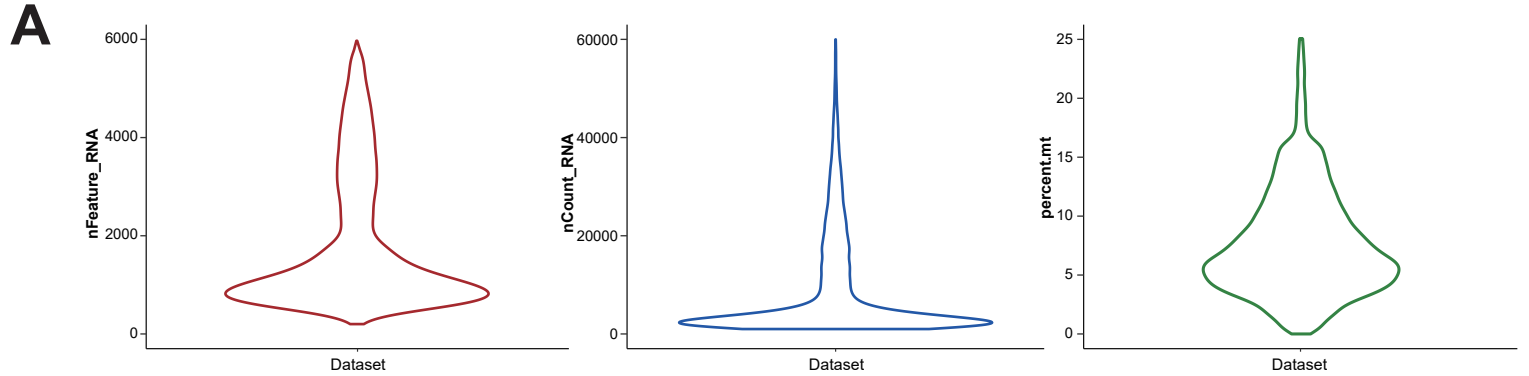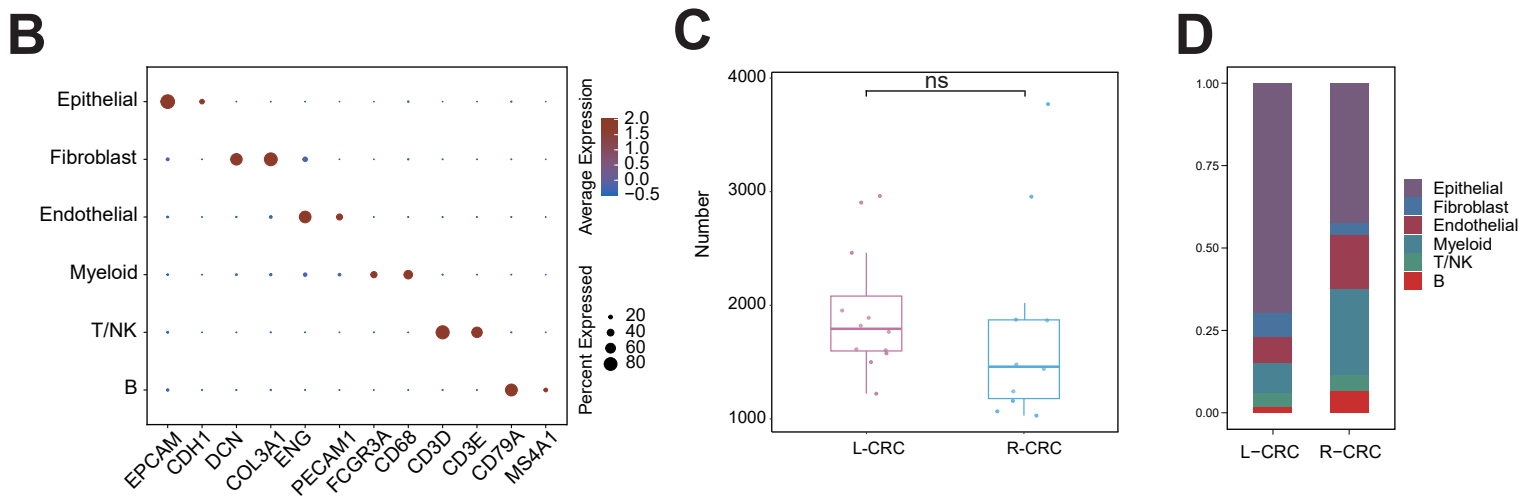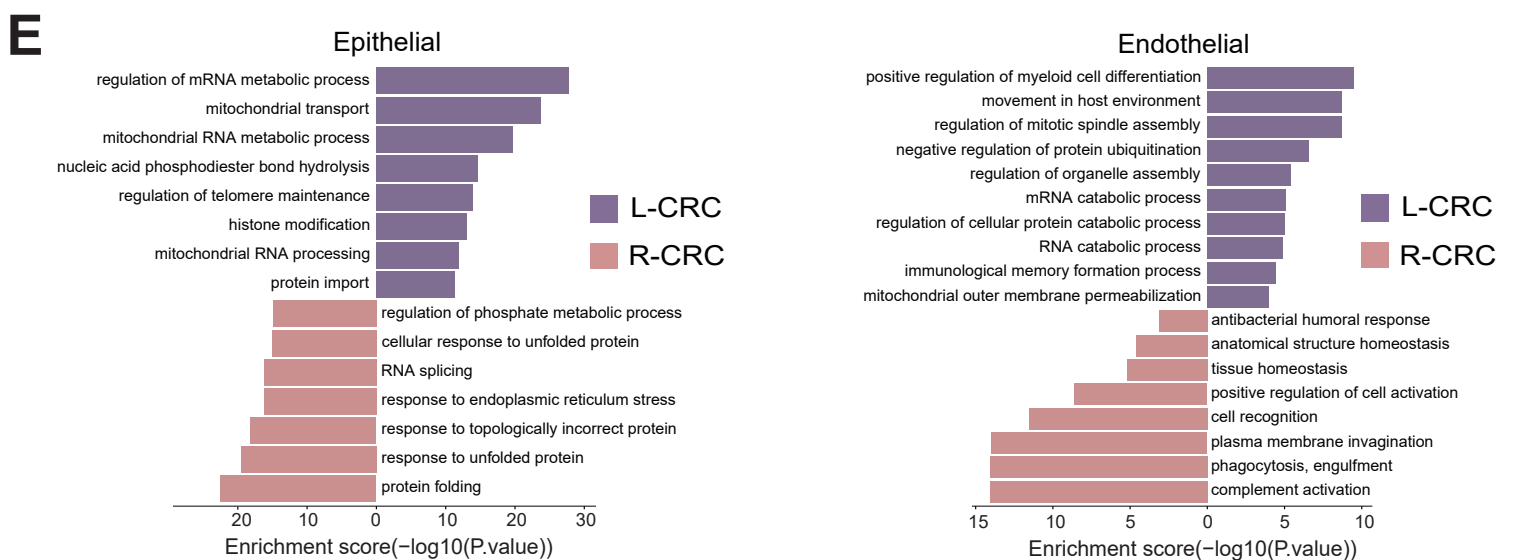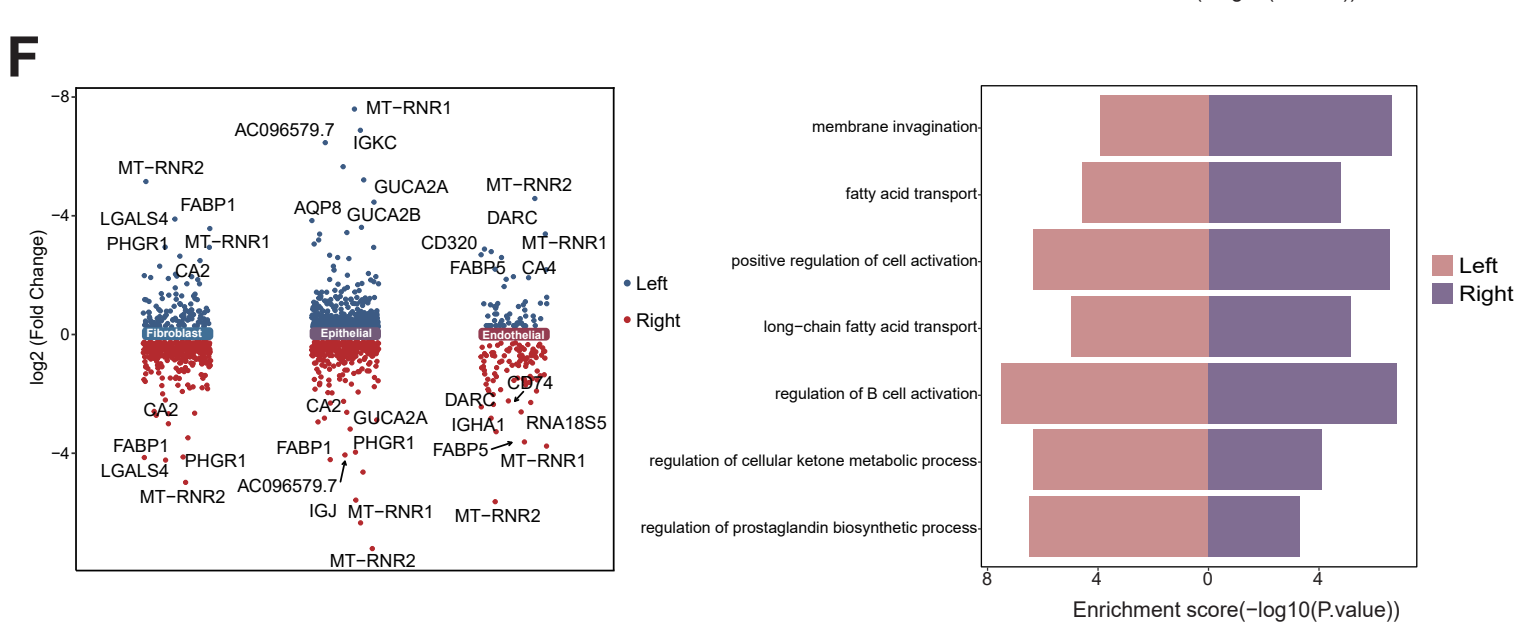

Supplement: Supplementary file 1 — Figure S1. [file JCMM-28-e70102-s001.pdf]

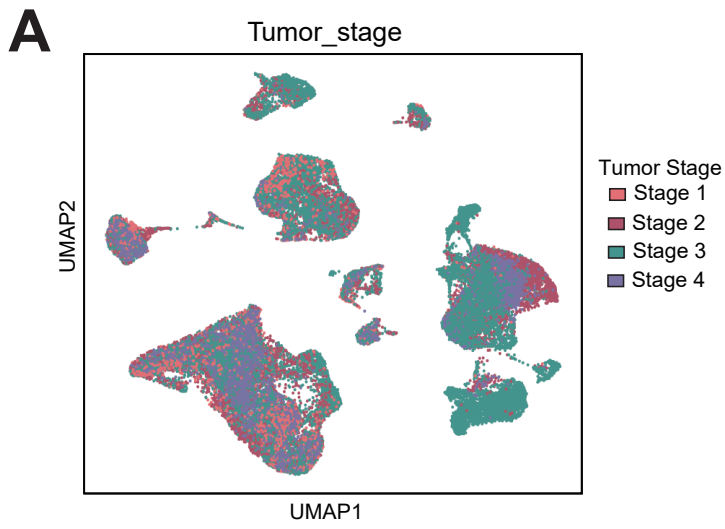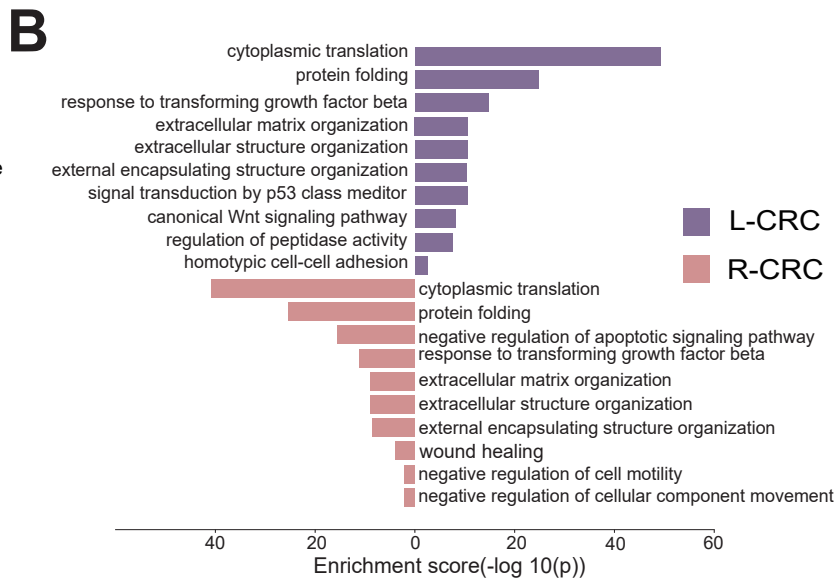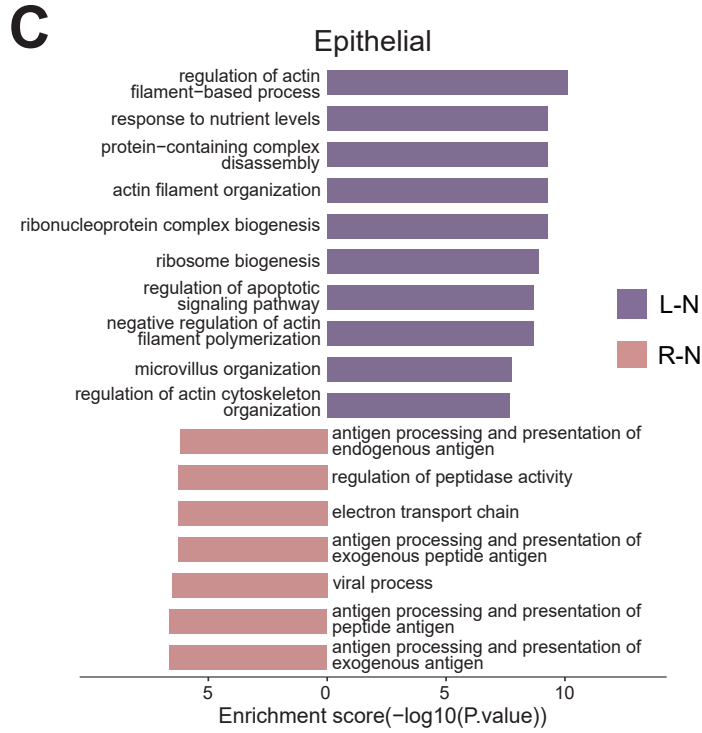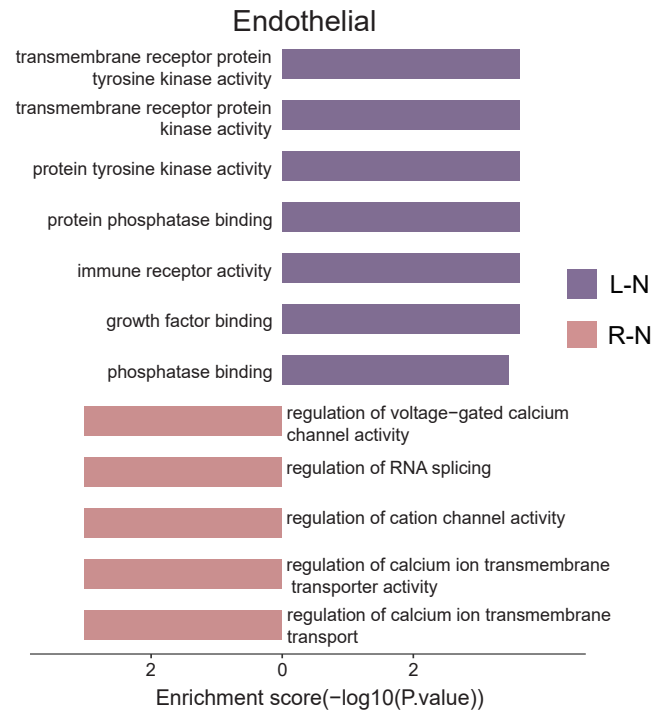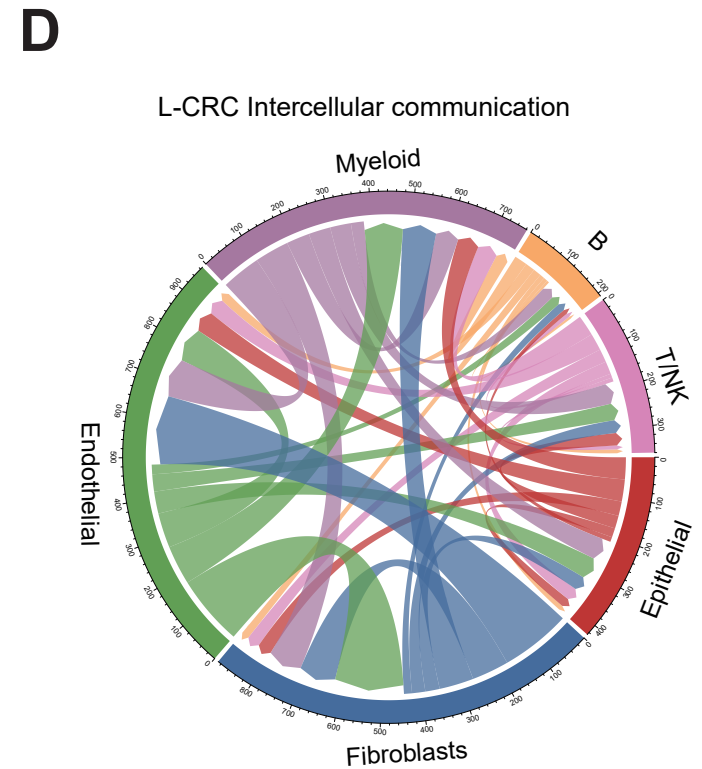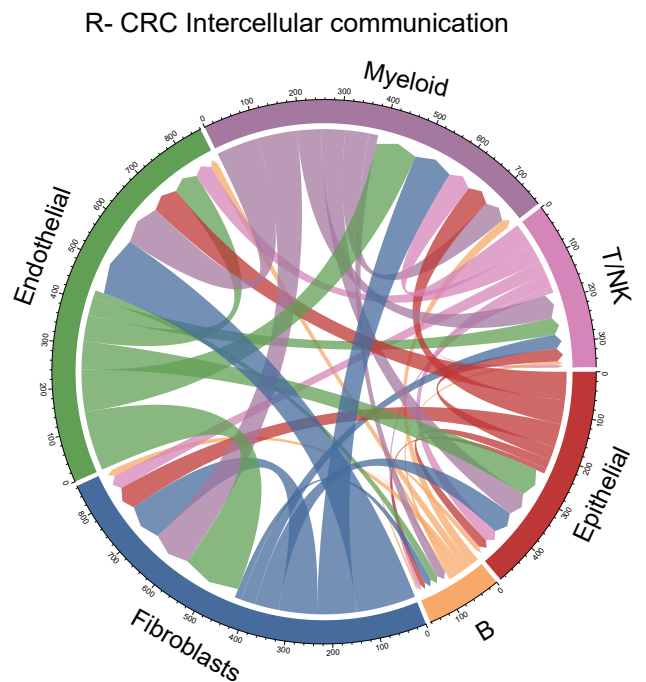

Supplement: Supplementary file 2 — Figure S2. [file JCMM-28-e70102-s005.pdf]
