# Supplementary material for: Single‐cell RNA sequencing reveals the heterogeneity of MYH11+ tumour‐associated fibroblasts between left‐sided and right‐sided colorectal cancer
Source: J Cell Mol Med. 2024 Sep 18;28(18):e70102. doi: 10.1111/jcmm.70102 (PMC11410558; doi:10.1111/jcmm.70102)
Supplement: Supplementary file 3 — Figure S3. [file JCMM-28-e70102-s004.pdf]

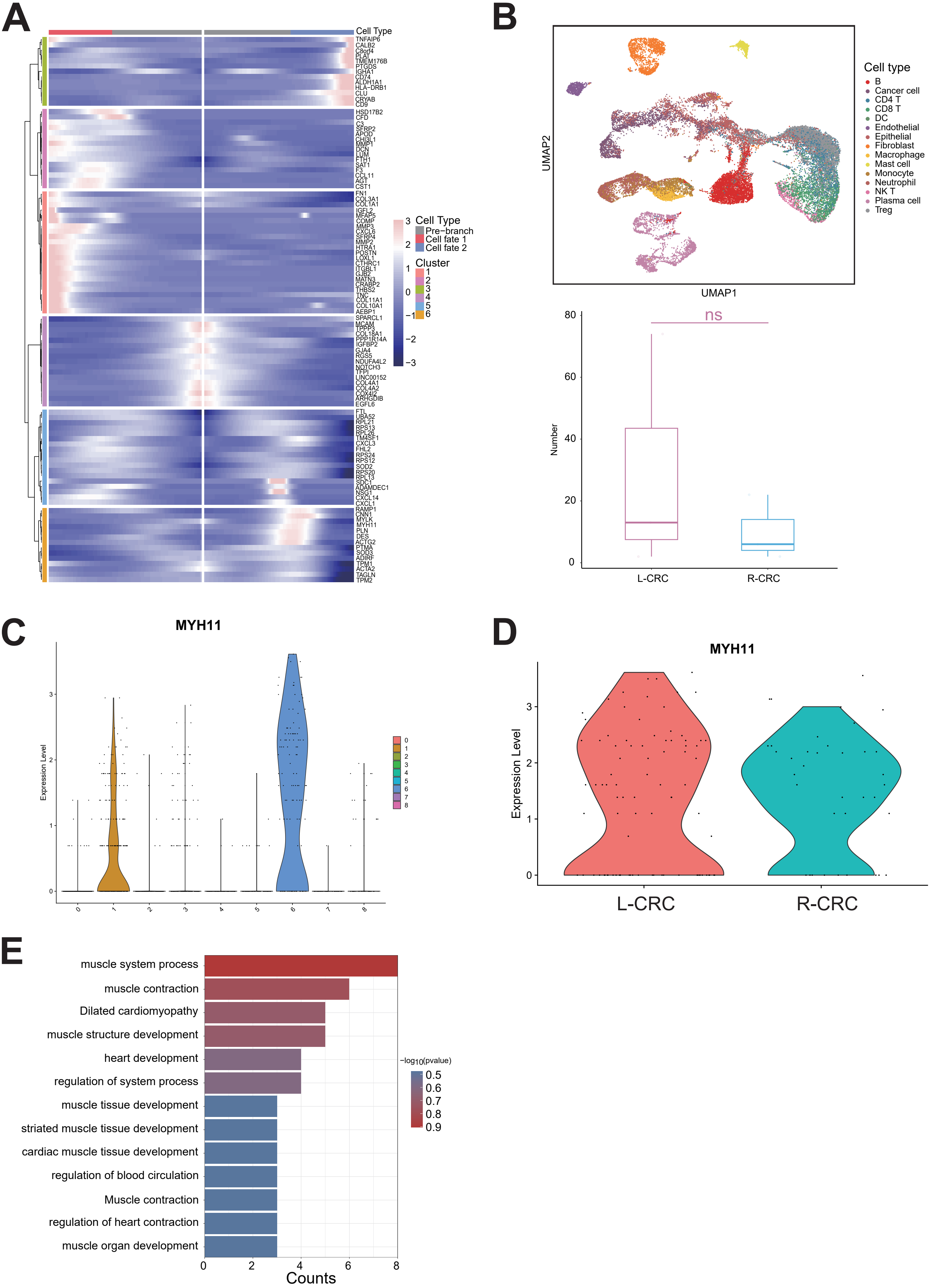
A
Cell Type
TNFAIP6
CALB2
C8orf4
PLAT
TMEM176B
PTGDS
IGHA1
CD74
ALDH1A1
HLA−DRB1
CLU
CRYAB
CD9
HSD17B2
CFD
C3
SFRP2
APOD
CHI3L1
MMP1
DCN
LUM
FTH1
SAT1
F3
CCL11
AGT
CST1
FN1
COL3A1
COL1A1
IGFL2
MFAP5
COMP
MMP3
CXCL6
SFRP4
MMP2
HTRA1
POSTN
LOXL1
CTHRC1
ITGBL1
GJB2
MATN3
CRABP2
THBS2
TNC
COL11A1
COL10A1
AEBP1
SPARCL1
MCAM
TPPP3
COL18A1
PPP1R14A
IGFBP2
GJA4
RGS5
NDUFA4L2
NOTCH3
TFPI
LINC00152
COL4A1
COL4A2
COX4I2
ARHGDIB
EGFL6
FTL
UBA52
RPL21
RPS13
RPL26
TM4SF1
CXCL3
FHL2
RPS24
RPS12
SOD2
RPS20
RPL13
SDC1
ADAMDEC1
NSG1
CXCL14
CXCL1
RAMP1
CNN1
MYLK
MYH11
PLN
DES
ACTG2
PTMA
SOD3
ADIRF
TPM1
ACTA2
TAGLN
TPM2
3
2
1
0
−1
−2
−3
Cell Type
Pre−branch
Cell fate 1
Cell fate 2
Cluster
1
2
3
4
5
6
B
UMAP2
UMAP1
Cell type
B
Cancer cell
CD4 T
CD8 T
DC
Endothelial
Epithelial
Fibroblast
Macrophage
Mast cell
Monocyte
Neutrophil
NK T
Plasma cell
Treg
ns
Number
80
60
40
20
0
L-CRC
R-CRC
C
MYH11
Expression Level
3
2
1
0
0
1
2
3
4
5
6
7
8
D
MYH11
Expression Level
3
2
1
0
L-CRC
R-CRC
E
muscle system process
muscle contraction
Dilated cardiomyopathy
muscle structure development
heart development
regulation of system process
muscle tissue development
striated muscle tissue development
cardiac muscle tissue development
regulation of blood circulation
Muscle contraction
regulation of heart contraction
muscle organ development
−log10(pvalue)
0.5
0.6
0.7
0.8
0.9
0
2
4
6
8
Counts
